# The Relation of Marital Adjustment and Family Functions With Quality of Life in Women

**DOI:** 10.5964/ejop.v11i3.859

**Published:** 2015-08-20

**Authors:** Sajjad Basharpoor, Ali Sheykholeslami

**Affiliations:** a Department of Psychology, University of Mohaghegh Ardabili, Ardabil, Iran; b Department of Educational Sciences, University of Mohaghegh Ardabili, Ardabil, Iran; Aalborg University, Aalborg, Denmark

**Keywords:** marital adjustment, functions, family, quality of life

## Abstract

Given the immense importance of marital relationships in the quality of life, this research was conducted in order to investigate the relationships between marital adjustment and family functions with quality of life in women. The design of the current study was correlational. Seven hundred and thirty women were selected randomly among all women living in the province of Western Azerbaijan (Iran) and participated in this study. The sample responded to the Family Assessment Device, Dyadic Adjustment scale and Quality of Life questionnaire, individually in their homes. Collected data were analyzed by Pearson’s correlation and multiple regression tests. The results showed that all dimensions of family functions and dyadic adjustment were positively correlated with quality of life in women. Results of multiple regression also revealed that 33 percent of total quality of life can be explained by family functions and 24 percent of this variable can be explained by dyadic adjustment. Our study demonstrated that women’s quality of life was affected by family functions and marital adjustment in family.

## Introduction

Following the World Health Organization’s definition of health as physical, mental and social well-being, more researchers goes beyond attending the absence of symptoms in their assessments of health and recovery from diseases. One of concepts introduced in this context which has been considered in various studies is quality of life. Quality of life is a holistic construct that views human health and well-being within the context of proximal and distal environments (Lindström, 1992). The World Health Organization defines quality of life as individuals’ perceptions of their position in life in the context of the culture and value system in which they live and in terms of their goals, standards, and concerns (WHOQOL Group, 1998). The definition includes six broad domains: physical health, psychological state, levels of independence, social relationships, environmental features, and spiritual concerns. This broad definition includes some aspects such as environment (housing, clothing, food etc.).

Trying to determine factors associated with people’s quality of life, researchers have revealed that the family and the interaction styles among its members is a prominent dimension related to people’s quality of life, happiness and well-being in all cultures. Studies have accumulated strong evidence showing that family life affects happiness greatly (Rodgers & Bachman, 1988). Chilman (1982) reviewed major national surveys from 1957 to the end of the 1970s and concluded that even though societal views of marriage and family have undergone dramatic and fundamental transformations, family life is still widely seen as central to life satisfaction and happiness; furthermore, married people have an advantage, on indices of anxiety and unhappiness, over those who are single, divorced or separated (Lu & Lin, 1998).

Low quality of life and marital distress co-occur frequently, particularly among women. Studying psychosocial variables associated with depression, Brown and Harris (1978) found that the lack of a confiding relationship is a vulnerability factor in the development of depression in women. Specifically, low intimacy with husband was associated with depression in women. In 1987, Weismann reported that individuals in unhappy marriages are 25 times more likely than those in happy marriages to be diagnosed with clinical depression. Examining the role of humiliating marital events, such as husband infidelity or threat of marital dissolution on the wife’s depressive symptoms, Cano and O’Leary (2000) found that after controlling for levels of marital discord, women who had experienced such severe marital stressors were six times more likely to be diagnosed with a major depressive episode. These findings remained even after controlling for lifetime and family histories of depression. Depressed individuals in unhappy marriages also recover less quickly from a depressive episode (e.g., McLean, Ogston, & Grauer, 1973), and are more likely to experience a relapse of their depressive symptoms (Fiedler, Backenstra, Kronmüller, & Mundt, 1998). Whisman (2001) found that marital dissatisfaction accounted for approximately 18% of the variance of wives’ depressive symptoms and 14% of husbands’ depressive symptoms. Gabriel, Beach, and Bodenmann (2010) found that arital interaction behavior depends on gender, depression and marital distress. Waite, Luo, and Lewin (2009) found general support for the hypothesis that emotional well-being tends to decline following marital disruption, across a range of dimensions of well-being. Darvizeh and Kahki (2008) reported that there are correlations between marital satisfaction and well-being in married female college students.

Regarding physical health, empirical examination indicates that marital quality and satisfaction are positively related to measures of global health (Hetherington, 1993), as well as indices of better immune (antibody titers to various viral agents; Kiecolt-Glaser et al., 1988) and cardiovascular system functioning (Ewart, Taylor, Kraemer, & Agras, 1991). Furthermore, a decline in marital satisfaction is associated with a decline in the self-reported health of both partners (Levenson & Gottman, 1985). Lastly, marital satisfaction is related to pronounced cardiovascular reactivity during conflict, such that unhappily married individuals display faster heart rates and greater elevations in blood pressure than happily married individuals (Ewart et al., 1991; Whitson & El-Sheikh, 2003).

Results of Giannouli et al. (2012) showed that higher total QOL in women could be predicted by being married, physical exercise and a good financial status. In this study, women with a better QOL were more health conscious and more likely to utilize the public health preventive resources. Results of Alayi, AhmadiGatab, and Khamen (2011) suggested that spousal compatibility of those couples with strong communication skills in various aspects of spousal relationship was significantly higher than those with weaker communication skills. Portes, Kyle, and Eaton (1992) found that the control of behavior and affective involvement plays an important role in facilitating socio-affective adjustment between couples. Bahari (2000) showed that distressed couples perform weakly in affect impression, family roles and problem solving. In the study of Assari et al. (2008), dyadic adjustment showed significant correlations with total scores of quality of life and with most of its sub-scores, Affective expression was significantly correlated with role limitations, social functioning, general mental health, vitality, general health perceptions, physical composite score (PCS) and mental composite score (MCS). Dyadic consensus was not correlated with any quality of life sub-scores. Trudel and Goldfarb (2010) found that Satisfying marital functioning protects against the development of psychological distress but is also a factor related to depression and anxiety, in this study the link between marital distress and depression is particularly strong. Results of Peterson-Post, Rhoades, Stanley, and Markman (2014) on married couples from the community during their first year of marriage and at three time points over the next 10 years, revealed that Initial marital adjustment predicts depressive symptoms for husbands and wives at all follow-ups significantly.

Overall, it appears that disruptions in the marital relationship and family functions may lead to weak quality of life, especially for women. Considering women’s health and well-being as the important factors related to family health and ultimately society’s health, the purpose of the current study is to investigate the relationships of marital adjustment and family functions with quality of life in women.

## Method

### Participants and Procedure

The design of the current study was correlational. All women living in Western Azerbaijan province in 2011 made up the statistical society of this research. Seven hundred and thirty women, selected randomly, participated in this study. It is necessary to note that the primary sample size of this study was 1000 women. However, at the time of data collection, 133 questionnaires were not returned and 137 questionnaires were answered by husbands, so, these questionnaires were excluded from the research.

### Measures

#### Demographic questionnaire

This questionnaire was administered to gather demographic characteristics like age, educational status, employment status, monthly income of family and religion. Collected data were used for descriptive purposes.

#### Family Assessment Device (FAD)

The FAD is a well-studied measure that uses a Likert type scale to assess family functioning. The measure consists of numerous scales, among which are problem solving, communication, roles, affective regulation, affective involvement, general family functioning, and behavior control. Though the subscales have demonstrated good internal consistency with Cronbach’s alpha ranging from .72 to .92 (Epstein, Baldwin, & Bishop, 1983), some have argued that overlap between 13 subscales warrants a complete reorganization of the measure (Ridenour, Daley, & Reich, 2000). Nonetheless, scores from the general family functioning scale and the behavior control scale are generally seen as measuring independent aspects of family functioning (Ridenour, Daley, & Reich, 2000).

#### Revised Dyadic Adjustment Scale (R-DAS) 

The R-DAS is a measure of dyadic adjustment based on the scale developed by Spanier (1976). The measure provides a total R-DAS score (range 0 - 69, α = .90), as well as scores for the following three subscales: consensus (α = .81), satisfaction (α = .85), and cohesion (α = .80) (Busby, Christensen, Crane, & Larson, 1995). Of particular interest for this study is the established cutoff of 48 for the total score, which allows a distinction to be made between distressed and non-distressed couples (Crane, Middleton, & Bean, 2000). RDAS has been previously widely used among Iranian subjects (Fathi-Ashtiani et al., 2007). In this study, Cronbach alpha was between 0.70 and 0.80 for the different subscales of RDAS.

#### World Health Organization Quality of Life Assessment-Brief (WHOQOL) (WHOQOL Group, 1998) 

The WHOQOL is a 26-items measure that assesses general quality of life. Questions ask respondents to rate how they function in the domains of Physical Health, Psychological Health, Social Relationships, and the Environment (e.g., the ability to function in one's physical environment). Subscale scores as well as an overall score are obtained. Items are rated on a 5-point Likert scale with different anchors for different questions. Previously reported Cronbach's alphas for the scale for a sample of women and men were as follows: [Physical Health *r* = 0.80; Psychological Health *r* = 0.76; Social Relationships *r* = 0.66; Environment *r* = 0.80]. According to the WHOQOL authors, the lower alpha for the Social Relationships scale is due to the fact that the scale comprises of only 3 items. Cronbach's alphas for the total score for the current sample were 0.90 for women and 0.91 for men. Test–retest reliability estimates for a combined sample of women and men were reported by the scale authors [Physical Health *r* = 0.66; Psychological Health *r* = 0.72; Social Relationships *r* = 0.76; Environment *r* = 0.87]. Validity has been demonstrated in the WHOQOL's ability to discriminate between individuals classified as ill and those classified as well (WHOQOL Group, 1998). Najatsafa, Montazeri, Holakuiy, Mohamad, and Majdzade (2006) found good reliability for all subscales of this questionnaire in Iranian subjects (physical health: *r* = 0.77, mental health: *r* = 0.77, social relationship: *r* = 0.75 and environment health: *r* = 0.84).

### Data Analysis

Collecting data just began after receiving permission from the local authorities to visit the homes of the participants sampled for the study. The purpose of the study was explained to the women and after obtaining written consent, they were asked to respond to the demographic questionnaire, Family Assessment Device, Dyadic Adjustment Scale and Quality of Life Questionnaire individually and in their house. Collected data were analyzed using descriptive statistics and the Pearson’s correlation and multiple regression tests.

## Results

Seven hundred and thirty women with the mean age (± *SD*) of 35.76 (± 9.66) participated in this research.

Table 1 shows the educational status of 43.1% of participations being under the diploma and 53.2% of them being between diploma and BA. 32.5% of participations were employed and 64.8% were unemployed. In terms of family income, 75.2% of participants had incomes ≥ 600$. 79.2% participations were SHIEE and 20.1% were SONNY in terms of religion.

**Table 1:** Descriptive Characteristic of Participants

Variable	Frequency	Percent
Educational status		
Under the diploma	315	43.2
Between diploma and BA	388	53.2
Between BA and MA	13	1.8
Not reported	14	1.9
Employment status		
Employee	237	32.5
Unemployed	473	64.8
Not reported	20	2.7
Family income		
Less than 600 dollar per month	549	75.2
Between 600 and 1000 dollar per month	107	14.7
Higher than 1000 dollar per month	47	6.4
Not reported	2	0.3
Religion		
SHIEE	578	79.2
SONNY	147	20.1
Other	3	0.4
Not reported	2	0.3

Table 2 results show that quality of life related positively to problem solving (*r* = 0.26; *p* < 0.001), communication (*r* = 0.23; *p* < 0.001), family roles (*r* = 0.37; *p* < 0.001), affective responsiveness (*r* = 0.29; *p* < 0.001), behavioral control (*r* = 0.16; *p* < 0.001), general function (*r* = 0.11; *p* = 0.01), dyadic consensus (*r* = 0.41; *p* < 0.001), dyadic expressions (*r* = 0.38; *p* < 0.001), dyadic satisfaction (*r* = 0.36; *p* < 0.001), dyadic cohesion (*r* = 0.17; *p* < 0.001) and the total score of dyadic adjustment (*r* = 0.49; *p* < 0.001).

**Table 2:** Pearson Correlations of Family Functions and Dyadic Adjustment With Quality of Life

Variables	M	SD	1	2	3	4	5	6	7	8	9	10	11	12	13
1. Problem solving	14.91	2.37	–												
2. Communication	14.75	2.89	.45***	–											
3. Family roles	20.74	3.34	.21***	.17***	–										
4. Affective responsiveness	13.46	2.20	.26***	.30***	.45***	–									
5. Affective involvement	21.87	4.30	.15***	.08*	.57***	.47***	–								
6. Behavior control	22.60	3.86	.24***	.26***	.53***	.47***	.52***	–							
7. General function	22.93	35.51	.07	.03	.15***	.04	.01	.08*	–						
8. Dyadic consensus	16.08	3.71	.25***	.22***	.23***	.01	.19***	.14***	.16***	–					
9. Dyadic expression	7.85	2.20	.27***	.20***	.19***	.04	.18***	.08*	.14***	.71***	–				
10. Dyadic satisfaction	13.51	4.14	.24***	.13***	.28***	.23***	.31***	.25***	.21***	.31***	.30***	–			
11. Dyadic cohesion	8.09	3.46	.24***	.07	.15***	.08**	.14***	.13**	.13**	.10**	.11**	.007	–		
12. Dyadic adjustment	45.67	8.72	.18***	.23***	.33***	.13**	.33***	.24***	.14***	.78***	.71***	.66***	.47***	–	
13. Quality of life	85.90	12.68	.26***	.23***	.37***	.14***	.29***	.16***	.11***	.41***	.38***	.36***	.17***	.49***	–

*p < .05. **p < .01. ***p < .001.

Table 3 shows that 33 percent of the total variance in quality of life is explained by family functions. The set of seven family functions as a whole contributed significant prediction to quality of life (*F* = 24.64, *p* < 0.001). Of the various indices of family functioning, only problem solving, communication and family roles are predictive of the quality of life.

**Table 3:** Multiple regression of quality of life on family functions

Predictors	*R*^2^	*F*	*p*	*B*	*B SE*	β	*t*	*p*	Collinearity statistics	
									tolerance	VIF
Model	0.33	24.64	< .001							
(Constant)				8.80	5.51		14.65	< .001		
Problem solving				1.48	0.28	0.26	5.21	< .001	0.77	1.28
Communication				0.93	0.22	0.21	4.26	< .001	0.77	1.29
Family roles				1.51	0.22	0.38	6.85	< .001	0.62	1.60
Affective responsiveness				0.12	0.30	0.02	0.41	.68	0.72	1.37
Affective involvement				0.28	0.17	0.09	1.63	.10	0.60	1.66
Behavior control				0.12	0.18	0.03	0.67	.50	0.67	1.49
General function				0.02	0.01	0.06	1.41	.15	0.93	1.06

Results of Table 4 show that 24 percent of the variance in quality of life is explained by marital adjustment. The set of dyadic adjustment variables totally contributed significant prediction to quality of life (*F* = 41.17, *p* < 0.001). However, the total score on dyadic adjustment scale was able to predict quality of life positively.

**Table 4:** Multiple Regression of Quality of Life on Dyadic Adjustment

Predictors	*R*^2^	*F*	*p*	*B*	*B SE*	β	*t*	*p*	Collinearity statistics	
									tolerance	VIF
Model	0.24	41.17	< .001							
(Constant)				52.94	2.69		19.63	< .001		
Dyadic consensus				0.12	0.25	0.03	0.47	.63	0.29	3.34
Affect expression				0.01	0.39	0.002	0.03	.96	0.42	2.34
Dyadic cohesion				0.32	0.18	0.08	1.73	.08	0.58	1.70
Total score of dyadic adjustment				0.83	0.12	0.56	6.65	< .001	0.21	4.77

## Discussion

Recently, the position of women in developing nations has been a subject of great interest to researchers (Prorok & Chhokar, 1998; Riley, 1998). considering the crucial role of the family in women's quality of life, this research was conducted in order to investigate the relationships of family functions and marital adjustment with quality of life in women. The results of Pearson’s correlation show that all functions of family and marital adjustment and its subscales were correlated with women's quality of life significantly. Consistent with previous researches (e.g., Alayi et al., 2011; Bahari, 2000; Brown & Harris, 1978; Chilman, 1982; Peterson-Post et al., 2014; Portes, Kyle, & Eaton, 1992; Rodgers & Bachman, 1988; Trudel & Goldfarb, 2010; Waite, Luo, & Lewin, 2009; Weissman, 1987), these results suggest that the family and quality of relations between its members are important factors which determine individual health and well-being. Furthermore, since women, especially in our society, are dependent on their families and husbands, the stability within the family affects their health greatly. The results of multiple regression also showed that family functions, especially problem solving, communication and family roles were able to significantly predict quality of life in women. It means that, women who solve family problems well, have good relations with other family members, perform family roles well and have adjustment with husbands, have a better quality of life. Corresponding to previous studies, such as those of Alayi et al. (2011), Bahari (2000), Brown and Harris (1978), Cano and O’Leary (2000), Peterson-Post et al. (2014), Waite et al. (2009), Weissman (1987), Whisman (2001), and Trudel and Goldfarb (2010) on relating family backgrounds of women to their quality of life, these results support the importance of the effect of the family context and family factors on people’s health and their quality of life.

In this study, functions such as affective responsiveness, affective involvement, behavior control and general function were significantly correlated with quality of life, but these functions could not predict the quality of life significantly. These results are not congruent with result of Portes, Howell, Brown, Eichenberger, and Mas (1992). It seems that in comparison to women, these family functions are more effective for children. Women attend more to how family members play their roles at home and how they solve problems that arise and what the quality of communication between family members is. Ultimately, these factors affect their health and quality of life.

Furthermore, another result of the present study was that marital adjustment was able to predict 24 percent of the total variance in women’s quality of life. This finding is also consistent with the results of Assari et al. (2008), Darvizeh and Kahki (2008), Hetherington (1993), Levenson and Gottman (1985), Peterson-Post et al. (2014), and Trudel and Goldfarb (2010) in relation to the role of marital adjustment in individuals’ quality of life. Based on scientific literature, it is apparent that characteristics of marriage, particularly marital adjustment have an impact on the health of individuals. Well-adjusted women have less conflict with their husbands and solve their problems easily, and so have good quality of life. Furthermore it can be claimed that marital maladjustment can weaken the immune system by creating stress and ultimately leads to low quality of life.

The limitations of this study should be shortly summarized. Due to the correlation design, causal relationships could not be identified; neither could the effect of family functions and marital adjustment on quality of life be evaluated. Furthermore, all parameters assessed were self-reported, introducing the possibility of inconsistencies. On the other hand, the large size of sample is the strength of this study.

### Conclusion

In conclusion, the results of this study demonstrated that family functions especially, problem solving, communications and family roles as well as marital adjustment can explain more than half of the quality of life in women. Therefore, it is suggested that any intervention in increasing women’s quality of life should take these aspects into consideration.
